# Association between dry eye and myopia in schoolchildren: current evidence and possible mechanisms

**DOI:** 10.3389/fmed.2026.1803506

**Published:** 2026-05-29

**Authors:** Hongsu Wang, Han Yu Zhang

**Affiliations:** 1 School of Medicine, Nankai University, Tianjin, China; 2 Institute of Optometry and Vision Science, School of Medicine, Nankai University, Tianjin, China

**Keywords:** dry eye, dry eye disease (DED), myopia, ocular surface, schoolchildren

## Abstract

The prevalence of myopia and dry eye disease (DED) has increased significantly in schoolchildren over the past few decades, particularly in East Asia, leading to intensive physical and emotional burdens on schoolchildren’s growth. Current studies reported associations between myopia and DED, where myopic individuals tend to have a higher prevalence of DED compared to non-myopic individuals among adults and children. The current review aims to summarize existing literature to investigate the relationship between DED and refractive error in schoolchildren. Possible mechanisms have been discussed, including mechanical and behavioral factors (ocular surface exposure, High-Order Aberrations, ethnicity, behavioral habits), parasympathetic dysregulation and neurovascular coupling.

## 1 Introduction

The prevalence of myopia has increased significantly over the past few decades, affecting approximately one-third of schoolchildren worldwide (1). The pandemic is particularly prevalent in East Asia, more than half of 19-years-old males affected in Seoul (2) and 75%–95% schoolchildren affected in Japan (3). It poses an even greater challenge in China. The overall myopia rate among Chinese schoolchildren remained at approximately 51.9%–53.6% during 2018–2022, which means more than 150 million schoolchildren are affected by myopia and we need to pay close attention to this issue (4).

Dry eye disease (DED) is another major public health concern and it may have a causal relationship with myopia. DED significantly contributes to financial burden on society (5). Among schoolchildren, DED not only causes physical discomfort but also significantly negatively impacts learning abilities, quality of life, and social functioning (6–8). Therefore, prompt diagnosis and treatment of DED are crucial for improving overall well-being. Numerous factors have been indicated to have contributed to DED in schoolchildren, including prolonged screen time (9), contact lens use (10), age (11), education level, and outdoor activity time (12).

Studies have shown that myopic individuals tend to have a higher prevalence of DED compared to non-myopic individuals in adults (13–19). A similar trend has been observed in schoolchildren. Studies in East Asian schoolchildren aged between 6 and 18 found that myopia individuals were more likely to suffer from DED (3, 20–22), and the association between refractive errors and signs or symptoms was statistically significant (*p* < 0.05) (3, 20). A strong association between myopia and DED has been suggested (23), where myopia may be a significant risk factor for DED among schoolchildren. This review aims to summarize existing literature to discuss the possible factors contributing to the association between DED and refractive error in schoolchildren.

## 2 Methodology

A comprehensive literature retrieval was executed across PubMed, Web of Science, and Embase, covering the period from January 2000 to May 2025. The search utilized keywords including “dry eye disease” “DED” “myopia” “dry eye and myopia” and “dry eye and myopia in schoolchildren.” Studies were selected based on their relevance to the pathophysiological interplay between refractive errors and ocular surface stability in schoolchildren. As a narrative review, this work prioritizes a conceptual synthesis of emerging mechanisms rather than a statistical meta-analysis. Therefore, while the PRISMA (Preferred Reporting Items for Systematic Reviews and Meta-Analyses) guidelines are primarily designed for systematic reviews, the core principles of search transparency and rigor were followed to ensure the quality of the literature selection process.

## 3 DED prevalence in adolescents with various refractive errors

In adolescents, the prevalence of DED demonstrates a positive correlation with the severity of myopia. Recent clinical studies consistently report a significant reduction in average tear break-up time (TBUT) and a higher incidence of symptomatic DED [diagnosed with Ocular Surface Disease Index (OSDI) score ≥13] in myopic adolescents compared to their emmetropic peers (3, 24, 25). A summary of recent clinical studies investigating the correlation between DED and myopia in schoolchildren populations is presented in Table 1. Specifically, high myopia serves as a pronounced risk factor, with prevalence rates reaching up to 1.5–2 times higher than in non-myopic individuals. This trend closely parallels the elongation of axial length (AL), suggesting an anatomical basis for the association (3, 24, 26).

**TABLE 1:** Overview of recent studies investigating the association between myopia and dry eye disease (DED) in schoolchildren.

Study title	Author (year)	Sample size and age	Key clinical findings	Proposed mechanisms and conclusions
Current prevalence of myopia and association of myopia with environmental factors among schoolchildren in Japan	Yotsukura et al. (3)	*n* = 1,478 Age: 6–15 y	DED symptoms positively correlated with refractive error severity (elementary) and AL (junior high).	Increased AL expands the exposed ocular surface area, accelerating tear film thinning and evaporation.
Questionnaire analysis on incidence and risk factors of dry eye in children from a myopia outpatient clinic	Wang et al. (20)	*n* = 214 Age: 10.1 ± 2.5 y	DED prevalence was 15.9%. Moderate myopia (−0.5D∼−3.0D) was significantly correlated with shorter NIBUT (*P* = 0.043).	Ametropia-induced visual fatigue alters tear film morphology, leading to reduced tear film stability (NIBUT).
Relation between dry eye and myopia based on tear film breakup time, higher order aberration, choroidal thickness, and axial length	Hazra et al. (25)	*n* = 72 Age: 12.8 ± 2.7 y	AL is negatively correlated with TBUT and positively correlated with higher-order aberrations.	A shared neuro-physiological link, possibly involving parasympathetic dysregulation, may underpin both conditions.
A possible reciprocal relationship between myopia and dry eye disease in Japanese teenagers	Ibrahim et al. (24)	*n* = 682 Age: 10–19 y	High myopes (≤−6.0D) showed the highest DED prevalence (34.9%) but reported lower pain sensitivity.	High myopia may impair ipRGC function, resulting in a “symptom-sign discordance” where objective dryness is severe but pain is underreported.
Prevalence of symptomatic dry eye and influencing factors among Chinese adolescents: a cross-sectional study	Chen et al. (26)	*n* = 1,518 Age: 12–18 y	High myopia (≤−6.0D) is an independent risk factor for symptomatic DED (*P* = 0.025).	The association may involve common autonomic nervous system pathways affecting both lacrimal secretion and choroidal thickness.
Relationship between myopia and diagnosis rates of dry eye disease: a systematic review and meta-analysis	Wu et al. (28)	14,232 eyes Mean age: 17.5 y	Myopes exhibit a 104% higher DED prevalence and significantly shorter TBUT vs. emmetropes.	Myopia is a potential risk factor for DED due to both structural ocular changes (e.g., AL) and behavioral usage patterns (e.g., blink frequency).

It is worth noting that while the association between myopia and DED is highly consistent in adolescent populations, research involving adult cohorts has yielded more complex findings. In adult demographics, some studies report that while mild-to-moderate DED is predominantly linked to myopia, severe DED is more frequently observed in hyperopic individuals (14). Furthermore, certain adult-based studies indicate a positive association between DED prevalence and the severity of hyperopia, rather than myopia (13, 15, 16, 27). This discrepancy suggests that the pathophysiological drivers of DED may shift with age; for instance, age-related accommodative strain may play a larger role in hyperopic adults, whereas structural changes like axial elongation are the primary catalysts in myopic youths (14, 28, 29). In conclusion, despite the heterogeneous findings in adult populations, current epidemiological evidence strongly supports that myopia–particularly high myopia accompanied by significant axial elongation–is unequivocally and positively associated with DED prevalence and severity in adolescents.

## 4 Possible mechanisms between myopia and DED

Overall, among adolescents aged between 10 and 18, myopia has been suggested to be strongly associated with increased prevalence of DED (3, 24–26). Multiple mechanisms have been proposed, which can be broadly categorized into four main domains: structural and anatomical alterations driven by axial elongation, behavioral habits, the parasympathetic nervous system (PNS), and retinal neurovascular coupling (NVC).

### 4.1 Structural and anatomical alterations: the axial elongation pathway

Increased ocular surface exposure and unstable tear film can increase the risk of DED (28). In adolescents with progressive axial myopia, where axial elongation occurs, there presents not only increased exposure area of ocular surface, resulting in a dry ocular surface environment, but also a changed corneal curve, leading to higher tension between the tear film and the cornea, thus leading to uneven distribution and hence accelerated evaporation of tear film (28, 30). Among patients with high myopia, the axial elongation of the eyeball may cause proptosis, which may contribute to DED symptoms following exposure keratopathy (31). Axial elongation leads to changes of the cornea, including flattening of the corneal curvature, reduced corneal thickness, and decreased endothelial density, which may contribute to reduced tear film stability and increased DED symptoms or clinical signs such as TBUT (17, 32). Additionally, because many myopic patients have prolonged use of electronic devices or near-distance reading, there is a significantly lower blink rate compared with that of the general population which may increase tear film evaporation and the risk of ocular surface dryness (28, 33, 34).

#### 4.1.1 The role of High-Order Aberrations (HOAs)

High-Order Aberrations act as a catalyst within a vicious pathophysiological cycle of DED and myopia. A stable pre-corneal tear film is essential for minimizing optical scattering. In patients with DED, the rapid break-up of the tear film disrupts the smooth anterior optical interface. This disruption acutely elevates both corneal and total ocular HOAs, drastically reducing retinal image quality (35–38).

This optical degradation establishes a critical link to myopia via the visual experience mechanism. Prolonged exposure to degraded retinal images, induced by DED-related HOAs, acts as a form of visual form deprivation. Such continuous blurred sensory input drives axial elongation and myopic progression (39, 40), plays an important role in AL growth among young animals (41–43) including humans (43–46).

**FIGURE 1:**
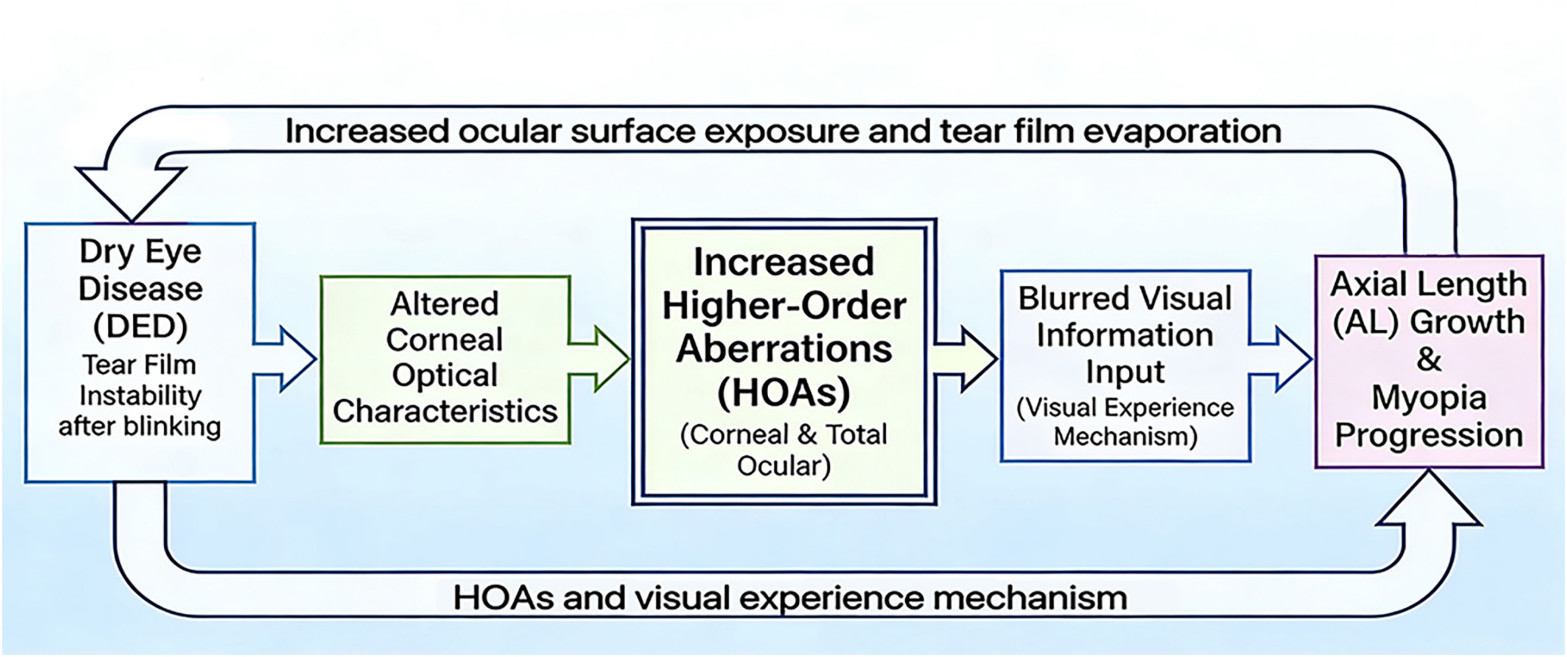
A hypothesized pathway illustrating High-Order Aberrations (HOAs) playing a role in the relationship between dry eye disease (DED) and myopia. DED may lead to altered corneal optical characteristics and increased HOAs due to increased tear film instability, which in turn contributes to axial elongation via visual experience mechanism and larger exposed ocular surface. This mechanically accelerates tear evaporation and thins the tear film, thereby exacerbating DED severity, consequently establishing a pathological cycle between axial elongation and ocular surface instability.

To address how these HOAs subsequently affect the severity of DED, one must consider the structural consequences of this myopic shift, which initiates a vicious pathophysiological cycle (Figure 1). As myopia progresses, the accompanied axial elongation enlarges the globe, which consequentially increases the palpebral fissure width and the exposed ocular surface area. A larger exposed ocular surface mechanically accelerates tear evaporation and thins the tear film, thereby exacerbating DED severity (3). Therefore, while DED-induced HOAs do not directly inflame the ocular surface, they indirectly worsen DED by driving structural axial elongation, forming a self-reinforcing loop between optical degradation and anatomical vulnerability (Figure 1). Previous studies focused mainly on adult populations (35–38) while fewer on adolescents (25, 26).

#### 4.1.2 The influence of ethnicity and genetic predisposition

Ethnicity serves as a critical background factor that modulates the axial elongation-mediated structural pathway described in Section “4.1 Structural and anatomical alterations: the axial elongation pathway.” TFOS DEWS III Report reported Asian ethnicity as a risk factor for DED development (47). Children as well as adults among studies conducted in Asia tend to have significantly shorter TBUT than those from other regions (48, 49). The genetic predisposition of East Asian schoolchildren to undergo early and rapid axial elongation (50) serves as a structural foundation that simultaneously drives myopic shift and accelerates the onset of exposure-related DED. Therefore, the high co-occurrence of these diseases in Asian populations is not merely coincidental but stems from a shared biomechanical vulnerability intrinsic to their ocular anatomy: the accelerated axial elongation inherently increases palpebral fissure width, thereby exacerbating the exposure-related tear evaporation mechanism introduced in Section “4.1 Structural and anatomical alterations: the axial elongation pathway.” Participants with Asian ethnicity have also been observed with meibomian gland shortening (51), incomplete blinking, lower lid epitheliopathy (52) and corneal astigmatism (53, 54), suggesting that ethnic variations significantly influence baseline AL and globe morphology, mirroring the anatomical mechanisms associated with axial elongation-induced surface exposure and subsequent tear evaporation with DED symptoms.

### 4.2 Behavioral habits and environmental factors

Behavioral habits may also play a role in the association between myopia and DED development. Sahai et al. proposed that individuals with refractive errors are more prone to eye rubbing, which can introduce foreign particles and destabilize the tear film, contributing to DED symptoms (15). Wang et al. (20) reported a significant relationship between eye rubbing, picky eating, and childhood DED in a cross-sectional study, attributing it to not only increased ocular surface inflammation following rubbing but also insufficient intake of omega-3 fatty acids and vitamin A, consistent with prior findings (55). Additionally, near work activity and screen time, as dominant risk factors of myopia (56), are significantly associated with a reduced blink rate, leading to increased tear evaporation and tear film instability. This may help explain the high prevalence of myopia and DED among school-aged children with prolonged screen time (9, 12, 23, 55, 57–60).

### 4.3 Parasympathetic nervous system

The parasympathetic nervous system (PNS) has been proposed as a potential upstream regulator for TBUT and choroidal thickness, which are respectively key objective measures for DED and myopia (25, 61, 62). Evidence suggested that preganglionic fibers from the superior salivatory nucleus project to the pterygopalatine ganglion (PPG), where postganglionic fibers simultaneously innervate the lacrimal gland and the choroid (63, 64). Ultrastructural studies have confirmed that fibers originating from the PPG utilize vasoactive intestinal polypeptide (VIP) as one of primary neurotransmitters to modulate these ocular structures (64).

**FIGURE 2:**
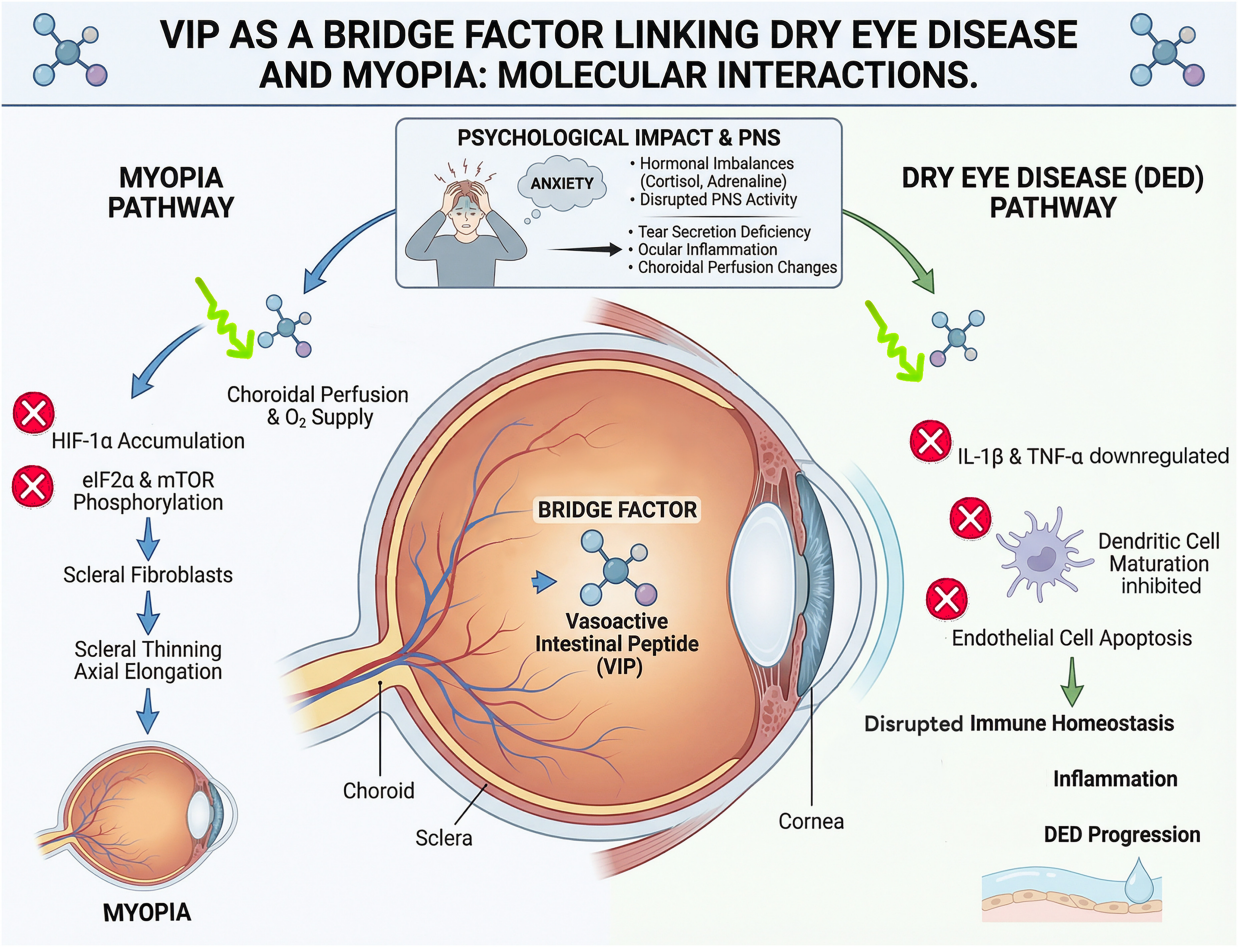
Vasoactive intestinal peptide (VIP) as a bridge factor between dry eye disease (DED) and myopia. VIP acts as a parasympathetic nervous system (PNS) neurotransmitter by dilating choroidal vessels to ensure oxygen supply and suppressing ocular surface inflammation. VIP reduction not only thins the sclera, but also breaks tear film homeostasis, which can respectively induce myopia and dry eye disease (DED), while anxiety-driven PNS inhibition further lowers VIP, exacerbating clinical signs and symptoms.

As illustrated in Figure 2, VIP serves as a critical biochemical bridge between DED and myopia. On the one hand, among myopes, VIP can increase choroidal perfusion pressure by its potent vasodilatory effects (63) to increase oxygen supply of the choroid and retina. If the level of VIP decreases, it may lead to decreased choroidal blood flow, causing an insufficient supply of oxygen. Scleral fibroblasts then respond to the altered micro-environment through the accumulation of Hypoxia-Inducible Factor 1α (HIF-1α) and enhanced phosphorylation levels of Eukaryotic Initiation Factor 2α (eIF2α) and Mammalian Target of Rapamycin (mTOR), which induces an increased fibroblast-to-myofibroblast transdifferentiation (FMT) and alterations in fiber diameter as well as a decrease in collagen production, resulting in a thinner and weaker sclera. Because the structural integrity of the sclera is compromised, it exhibits increased scleral creep and loses its resistance to normal intraocular pressure, leading to the passive expansion of the globe (65, 66). As a result, axial length increases, and myopia progresses (30, 67). On the other hand, for DED, VIP can exert potent anti-inflammatory effects by downregulating pro-inflammatory cytokines (such as IL-1β and TNF-α) (68, 69), inhibiting the maturation of dendritic cells (DCs) and promoting apoptosis over necrosis of dying endothelial cells (70, 71), thereby preventing immune-mediated damage to the corneal endothelium (72–74) and reducing the risk of DED progression. Hence, when there is a reduction of VIP, the ocular surface immune homeostasis may be compromised. Through those two pathways, DED and myopia are associated and affected synchronously:

1. Ocular Surface Impairment: Reduced VIP levels diminish the neuro-protective and anti-inflammatory capacity of the corneal environment, leading to tear film instability and the development of DED.
2. Scleral Hypoxia and Remodeling: Lowered VIP activity leads to choroidal vasoconstriction and subsequent thinning. The resulting scleral hypoxia acts as a potent trigger for axial elongation, thereby accelerating myopic progression.

Additionally, psychological factors such as anxiety, which are more prevalent among myopic adolescents (75, 76), may be a factor of the vicious cycle of DED and myopia. Anxiety often disrupts sleep patterns, leading to elevated cortisol and adrenaline levels while suppressing PNS activity (77). From a mechanistic perspective, anxiety-driven PNS inhibition leads to a systemic reduction in VIP levels. This depletion results in a “dual-pathway” pathology mentioned above. Consequently, the PNS does not merely act as a passive regulator but functions as an integrated signaling hub, where reduced VIP signaling serves as a common denominator for both conditions.

### 4.4 Retinal neurovascular coupling (NVC) dysfunction and ipRGC-mediated reflex tearing in high myopia

Emerging evidence points to a shared neural reflex arc–specifically mediated by intrinsically photosensitive retinal ganglion cells (ipRGCs)–that may bridge posterior neurovascular alterations with anterior surface homeostasis.

Studies revealed that the risk of DED increased with myopia severity, identifying high myopia as a risk factor of DED development (24, 26). Interestingly, while people with high myopia presented more dryness, significantly less pain and photophobia were reported compared to emmetropes and hyperopes (24). Progressive axial elongation in high myopia induces mechanical stretching and subsequent retinal neurovascular coupling (NVC) dysfunction, leading to localized metabolic imbalances in the posterior pole (24, 78, 79). This structural and vascular degradation has been shown to compromise the functional integrity of ipRGCs, as evidenced by diminished responses in chromatic pupillometry among highly myopic eyes (80).

Beyond their established role in non-image-forming vision, ipRGCs function as essential afferent sensors within the trigeminal-parasympathetic reflex arc, which regulates basal tear secretion and photophobia. We hypothesize that when retinal NVC dysfunction impairs ipRGC activity, this reflex loop is consequently blunted. Such an impairment may reduce neural-driven reflex tearing, exacerbating the objective signs of DED. Furthermore, this compromised neural pathway provides a mechanistic explanation for the clinical observation that highly myopic teenagers often report fewer DED symptoms–such as pain and photophobia–despite exhibiting severe tear film instability (24). Therefore, retinal NVC dysfunction in myopia may act not merely as an isolated posterior event, but as an upstream contributor to anterior ocular surface disruption via these compromised reflex arcs. The prevalence of high myopia in adolescents and schoolchildren is becoming higher (81). Thus, identifying this integrated neurovascular-immunological axis is essential for understanding the relationship between DED and myopia.

## 5 Discussion

A critical question remains whether myopia acts as an independent risk factor for DED or if both conditions represent co-manifestations of shared behavioral patterns. Undoubtedly, shared environmental catalysts–most notably the global surge in Digital Eye Strain (DES) and Computer Vision Syndrome (CVS)–play a significant role (82). As established in the TFOS DEWS II (83) and further emphasized in the TFOS DEWS III (47), prolonged screen exposure and intensive near-work activities serve as dual-drivers. Specifically, these habits trigger chronic accommodative stress, as explored in Section “4.2 Behavioral habits and environmental factors,” which may lead to PNS dysregulation (84). This neural shift simultaneously facilitates axial elongation and compromises the efferent signaling for lacrimation, characterizing the two conditions as co-manifestations of autonomic imbalance. However, the anatomical and neurological sequelae of myopia likely exert an independent risk effect. Physically, as discussed in section “4.1 Structural and anatomical alterations: the axial elongation pathway,” axial elongation increases the ocular surface exposure area and accelerates tear evaporation, a correlation evidenced by significantly lower NIBUT scores in children with higher myopic refractive power, as shown in Section “4.1 Structural and anatomical alterations: the axial elongation pathway.” Furthermore, as discussed in Section “4.3 Parasympathetic nervous system,” pathological stretching of the retina in high myopia may damage intrinsically photosensitive retinal ganglion cells (ipRGCs). Since these cells are essential mediators of the light-driven lacrimation reflex, their impairment suggests that myopia directly diminishes the eye’s homeostatic capacity for tear production. Therefore, while behavioral habits are undeniable catalysts, the anatomical and neurological sequelae of myopia likely exert an independent risk effect on schoolchildren DED development.

For the classification of myopia, most studies included in this review defined myopia by SE rather than axial length, which may limit our understanding of whether AL change solely contributes to DED pathogenesis like the mechanism in Section “4.1 Structural and anatomical alterations: the axial elongation pathway” or refractive error itself may influence DED in some unknown certain mechanism. Although axial myopia is more common in East Asian children, a substantial proportion of myopic children presented with minimal axial elongation (85). Additionally, even emmetropic eyes continue to grow longer during childhood, so a simple arithmetic conversion from axial elongation to myopic shift often misses the mark in individual children (86).

Contrasting findings have been reported regarding the relationship between axial elongation and corneal and total HOAs (3, 25, 87–90). Most studies considered total HOAs (3, 87–90) while a 2022 study (25) classified HOAs into corneal, internal, and total HOAs (91). It was identified that corneal HOAs may be the most associated risk factor for myopia progression and axial elongation in elementary school students, while Hazra et al. (25) found that it is internal HOAs but not corneal HOAs among children with DED (TBUT <10s). Future studies are required to investigate different groups of HOAs, in order to confirm a positive relationship with axial elongation and DED.

It is also important to note that varying criteria have been used for DED diagnosis among studies reported in this review. Some studies relied on subjective symptom questionnaires (e.g., OSDI, DEQ, SPEED), while others used objective clinical tests (e.g., TBUT, Schirmer’s test, and ocular staining). The use of the former offers the advantages of speed, simplicity, and high patient compliance; however, their results are strongly influenced by individual pain thresholds, cultural background, and psychological status. On the other hand, objective tests provide reproducible and quantifiable indices. The relationship between symptoms and signs of DED is not always linear and can vary among individuals with different DED subtypes (92, 93). For instance, due to retinal NVC dysfunction, individuals with high myopia, on the contrary, reported less pain and photophobia. The TFOS DEWS III report acknowledged the significance of both signs and symptoms in the diagnosis of DED and recommended a standardized diagnostic criteria for DED that is essential for research and clinical management (94).

A major limitation in the current literature is the heterogeneity of DED diagnostic criteria, which frequently rely on adult-centric standards that are poorly adapted for schoolchildren populations. As far as is known, few clinical guidelines provide distinct, validated diagnostic cut-off values specifically for children. Consequently, researchers and clinicians often extrapolate adult thresholds to pediatric cohorts, which raises significant concerns regarding validity and reliability. Subjective questionnaires, such as the OSDI, are of limited validity in children as they evaluate activities irrelevant to youth (e.g., driving) and depend heavily on the child’s cognitive comprehension to articulate ocular discomfort. Similarly, objective evaluations like Schirmer’s test often yield poor reliability and high inter-examiner variability due to limited patient cooperation and pronounced reflex tearing during invasive testing. This methodological gap frequently manifests as pronounced symptom-sign discordance; children may exhibit objective signs of severe ocular surface damage while underreporting symptoms, or conversely, present with frequent blinking and eye-rubbing without explicitly articulating “dryness” (84, 85). For instance, as discussed in Section “4.3 Parasympathetic nervous system” regarding neurovascular coupling, individuals with high myopia often report paradoxically less ocular pain despite severe clinical signs. To address these inconsistencies, the TFOS DEWS III report emphasizes the imperative need for standardized, age-appropriate diagnostic criteria for adolescents to ensure accurate clinical assessment (47).

Finally, as most of the current studies are cross-sectional, future prospective longitudinal studies are required to delineate the dynamic relationships among DED symptoms, nerve structure changes, and myopia progression.

Furthermore, understanding these shared mechanisms highlights the necessity for integrated clinical management. Interventions must be carefully individualized. For instance, while optical treatments such as orthokeratology (OK lenses) are highly effective for myopia control, they place additional mechanical stress on the ocular surface and may be contraindicated for schoolchildren with severe, concurrent DED. Conversely, targeting shared behavioral and environmental factors, such as strictly managing digital screen time, minimizing eye-rubbing and maintaining beneficial light signaling for myopia inhibition, offers beneficial and non-invasive strategies. Future longitudinal clinical trials evaluating these combined optical and behavioral interventions are warranted to determine their efficacy in restoring neuro-sensory homeostasis for both DED and myopia control.

This review highlights the relationship and possible causal mechanisms between myopia and DED among schoolchildren. Several assumed influence factors or mechanisms in this review include mechanical and behavioral factors (ocular surface exposure, High-Order Aberrations, ethnicity, behavioral habits), parasympathetic dysregulation and neurovascular coupling in high myopia. Limitations suggest that standardized research methodologies are needed to draw more objective definitive conclusions. Future longitudinal studies can focus on neurological mechanisms, environmental effects, and the role of HOAs to better understand how DED and myopia interact among adolescents and school-aged children.
